# Public interest in Cannabis during election season: a Google Trends analysis

**DOI:** 10.1186/s42238-020-00039-9

**Published:** 2020-09-22

**Authors:** Trevor Torgerson, Will Roberts, Drew Lester, Jam Khojasteh, Matt Vassar

**Affiliations:** Office of Medical Student Research, Oklahoma State University Center for Health Sciences, 1111 West 17th Street, Tulsa, OK 74107 USA

**Keywords:** Cannabis, Medical marijuana, Google trends, Elections, Politics, Internet search

## Abstract

**Introduction:**

Given that 72% of internet users seek out health information using an internet search engine (Google being the most popular); we sought to investigate the public internet search interest in cannabis as a health topic when cannabis legislation appeared on state ballots and during presidential elections.

**Materials and methods:**

We searched Google Trends for “cannabis” as a health topic. Google Trends data were extracted during the time period of May 1, 2008 to May 1, 2019 for the United States (US) and select states (18) within the US including: Alaska, Arizona, Arkansas, California, Colorado, Florida, Maine, Massachusetts, Michigan, Missouri, Nevada, North Dakota, Ohio, Oregon, Oklahoma, South Dakota, Utah, and Washington when cannabis was on the ballot. These state elections were referenda, not legislative votes. We then compared the internet search interest for cannabis before and after each election. To evaluate whether any associations with changes in the volume of cannabis internet searches were specific to the cannabis topic, or also occurred with other topics of general interest during an election year, the authors ran additional analyses of previously popular debated policies during Presidential Elections that may act as control topics. These policies included Education, Gun Control, Climate Change, Global Warming, and Abortion. We used the autoregressive integrated moving average (ARIMA) algorithm to forecast expected relative internet search interests for the 2012 and 2016 Presidential Elections. Individual variables were compared using a linear regression analysis for the beta coefficients performed in Stata Version 15.1 (StataCorp).

**Results:**

Public internet search interest for “cannabis” increased during the voting month above the previous mean internet search interest for all 18 bills. For the US, observed internet search interest during each Presidential Election was 26.9% [95% CI, 18.4–35.4%] greater than expected in 2012 and 29.8% [95% CI, 20.8–38.8%] greater than expected in 2016. In 2016, significant state-level findings included an increase in relative internet search rates for cannabis in states with higher usage rates of cannabis in the past month (Coeff (95% CI), 3.4 (2.8–4.0)) and past month illicit drug use except cannabis rates (Coeff (95% CI), 17.4 (9.8–25.0)). Relative internet search rates for cannabis from 2008 to 2019 were also associated with increased cannabis usage in the past month (Coeff (95% CI), 3.1 (2.5–3.7)). States with higher access to legal cannabis were associated with higher relative internet search volumes for cannabis (Coeff (95% CI), 0.31 (0.15–0.46)). Of the five additional policies that were searched as topics, only two showed an increase in internet search interest during each Presidential Election. Climate Change increased by 3.5% [95% CI, − 13-20%] in 2012 and 20.1% [95% CI, 0–40%] in 2016 while Global Warming increased by 1.1% [95% CI, − 19-21%] in 2012 and 4.6% [95% CI, − 6-15%] in 2016.

**Conclusion:**

Based on these results, we expect public interest in cannabis will spike prior to the Presidential election in 2020. Of the five selected control policies, only two showed an increase in internet search interest during both Presidential Elections and neither exceeded the internet search increase of cannabis. These results may indicate the growing awareness of cannabis in the US and mark a possible target for the timely dissemination of evidence-based information regarding cannabis and its usage/side-effects during future elections. Consequently, the results of this study may be important to physicians since they will likely receive an increased volume of questions relating to cannabis and its therapeutic uses during election season from interested patients. We recommend establishing a cannabis repository of evidence-based information, providing physician education, and a dosing guide be created to enable physicians to provide high quality care around the issue of cannabis.

## Introduction

In 2016, 22.2 million Americans over the age of 12 reported having used cannabis in the last 30 days (National Academies of Sciences, Engineering 2017). Cannabis continues to spark political debate regarding legalization for medicinal and recreational purposes (National Institute on Drug Abuse 2019). The potential economic and public health ramifications make legalization a polarizing issue. Healthcare, mental health, and addiction have been highly influential topics among presidential debates, of which, cannabis is only increasing in public awareness. Within healthcare specifically, cannabis has been discussed as an alternative therapeutic option to reducing the prevalence of opioid prescription use, including the abuse of these drugs (Wen and Hockenberry 2018; The October democratic debate transcript 2019).

The majority of ballot measures regarding adult use of recreational and medicinal cannabis have been included in the same voting cycle as presidential and midterm elections; however, the effect these elections have on public awareness of cannabis as a health topic is not yet known. Given that 72% of internet users seek out health information using an internet search engine (Fox and Duggan 2013); we sought to investigate the public internet search interest in cannabis as a health topic when cannabis legislation appeared on state ballots and during the presidential elections. We also aim to examine if states with higher increases in relative internet search rates for cannabis were associated with greater cannabis and illicit drug use the month before the 2016 election. Finally, we investigate state-level associations between relative internet search rates for cannabis and cannabis use statistics from the National Survey on Drug Use and Health.

## Materials and methods

On June 26, 2019, one author, (TT) searched Google Trends (https://trends.google.com/trends/?geo=US) for “cannabis” as a health topic and the relative-search volume (RSV), which is a query share for a particular time series, was then measured (Nuti et al. 2014). Through Google Trends’ search algorithm, search words can be defined as terms or topics depending on specific search needs. Search terms show matches for all terms in the query in the language given, while topics are a group of terms that share the same concept in any language. For example, when searching the term “banana,” results include terms like “banana”, “banana sandwich”, or “Banana Republic”. However, if the topic “London,” is searched results include terms categorized under the said topic such as “Capital of the UK” and “Londres,” which is “London” in Spanish (Compare trends search terms - trends help 2020). Therefore, searching for cannabis as a topic would allow the authors to encompass search words that fall under the cannabis category such as marijuana, medical marijuana, and recreational cannabis. Thenceforth, the *Google Trends* application allowed the authors to produce a series of time-trends graphs quantifying cannabis-specific online activity during the time of the election. This method is similar to previous studies looking at drug-legislative changes (Bright et al. 2013; Forsyth 2012). To evaluate whether changes in the volume of cannabis internet searches were specific to cannabis alone, or if these changes occurred with other topics of general interest during an election year, the authors ran additional analyses of previously popular debated issues during Presidential Elections that may act as control topics. These issues included Education, Gun Control, Climate Change, Global Warming, and Abortion. Prior to beginning the author’s internet search, the computer’s cache, cookies, and data were cleared on the Google Chrome browser to ensure that previous internet searches would not influence their results. “Cannabis”, and the additional control topics, were extracted from Google Trends data during the time period of May 1, 2008 to May 1, 2019 for the United States (US). Then, using the same date range, relative internet search data for “cannabis” was extracted for selected states (18) within the US including: Alaska, Arizona, Arkansas, California, Colorado, Florida, Maine, Massachusetts, Michigan, Missouri, Nevada, North Dakota, Ohio, Oregon, Oklahoma, South Dakota, Utah, and Washington when cannabis was on the ballot (Marijuana on the ballot - Ballotpedia 2019). These state elections were referenda, not legislative votes.

For each state election, the mean internet search interest for “cannabis” was obtained from May 1, 2008 through the month before each specific state election. This mean internet search interest was then compared to the internet search interest for “cannabis” during the state election month and cannabis use characteristics for the prior year. For example, the election date in Alaska occurred in November 2014, therefore we compared the mean internet search interest from May 1, 2008 to October 31, 2014 with the mean internet search interest during November, 2014. Only bills relating directly to the legalization of medicinal or recreational cannabis were included. When two bills were voted on for the same state within the set time period, the most recent ballot was used for analysis. Google Trends data is displayed as a relative search volume. The search volume for a given month over a prespecified time period (May 12,008 to October 31, 2014 in our example above) is given a relative search volume for each month ranging from 0 to 100. The relative search volume number for each month represents the search interest for a topic (cannabis in this study) relative to the month with the highest number of searches for the topic within the given time period. The relative search volume of 100 represents the upper limit and is the topic’s highest moment of popularity during that search period. All other monthly relative search volumes within the timeframe are displayed relative to the upper limit. For example, a relative search volume of 50 means there were half as many searches that month, compared to the highest number of searches within the timeframe.

Additionally, authors extracted state specific internet search rates for cannabis. To obtain the relative internet search rates for each state Google takes the total internet searches for a specific phrase originating from each state and divides it by the total number of Google searches from that state over the prespecified time frame. Similar to above, this relative internet search rate is then scaled from 0 and 100 relative to the state with the highest percentage of google searches for the topic within the given timeframe. This scaled search volume is referred to as relative search rate. Relative search rates for cannabis were obtained for each state in two separate time periods, May 1, 2008 to May 1, 2019 and the year of 2016. Each time period was used for specific analyses as outlined below.

In 2018, Americans for Safe Access evaluated each state’s medical cannabis laws in what they called “A Patient-Focused Analysis of the Patchwork of State Laws” (Reports [Internet] 2020). Americans for Safe Access graded state’s on their state-level access to legal cannabis. This access to legal cannabis score was based on factors from 5 categories: patient rights and civil protections, access to medicine, ease of navigation, functionality and consumer safety, and provider requirements (Reports [Internet] 2020). Each category is worth 100 points based on many specific qualifications. States’ grades were then calculated as a percentage of the 500 possible points. States without any form of medical cannabis laws at the time were not awarded grades (Idaho, Kansas, Nebraska, and South Dakota). Excluding the states without cannabis laws, we used specific relative search rates from the remaining 46 states and compared them with statistics from the National Survey on Drug Use and Health for 2016–17 for persons over the age of 18 years old (Comparison of 2015-2016 and 2016-2017 NSDUH population percentages 2020). State specific cannabis internet searches from 2016 were compared with cannabis use in the past month (18+ years old), illicit drug use in the past month excluding cannabis use (18+ years old) and access to legal cannabis grade. Each association was based on 46 pairs of data. State specific internet searches for cannabis from 2008 to 2019 were compared with “any illicit drug use in the past month (18+ years old)”. Comparatively, each control variable (Education, Gun Control, Climate Change, Global Warming, and Abortion) was also evaluated alongside “cannabis use in the past month (18+ years old)” and illicit drug use excluding cannabis in the past month (18+ years old) from the National Survey on Drug Use and Health for 2016–17 using the same methods outlined above. Individual variables were compared using a linear regression analysis for the beta coefficients performed in Stata Version 15.1 (StataCorp).

An autoregressive integrated moving average (ARIMA) algorithm was used to forecast expected relative internet search volumes for the 2012 and 2016 Presidential Elections. Data from Google Trends for the entire United States from May 1, 2008 to May 1, 2019 was extracted and used for this model The ARIMA model is based on time series analysis and prediction through a combination of seasonal variables. To start, all data was converted into a time series according to the year and month format, and was divided into two parts: pre-election and post-election for both 2012 and 2016. The pre-election data was used as model data and the post-election data was used as comparison data. Finally, the ARIMA model provides a predictive forecast for internet search queries had the elections not occurred (Liu et al. 2018; Liu et al. 2016). Additionally, in the event an increase in relative internet search volume occurred during the Presidential Elections of 2012 and 2016 we compared the mean internet search volume 6 months before each election and the mean internet search volume 6 months after each election to determine if the increase was momentary or sustained. R version 3.2.1 (R Foundation) was used for the ARIMA model and Stata Version 15.1 (StataCorp) was used for all statistical analyses.

## Results

Eighteen states and 18 total bills were included for analysis. Public internet search interest for cannabis increased for all 18 bills during the election month compared to rates preceding the election month. Across all 18 bills, the mean percent increase for internet search interest during the voting month was 48.1% [95% CI, 40.4–55.7%]. A complete depiction of results is outlined in Table 1.

**Table 1:** Public internet search interest in Cannabis legalization when Cannabis is on the Ballot

State	Election date	Medical or recreational	Pre-election internet search interest ^**a**^ [95, CI]	Increase ^**b**^ [95, CI]
Alaska	Nov 2014	Recreational	36.2 [34.5–37.9]	63.8% [62.1–65.5]
Arizona	Nov 2016	Recreational	56.5 [53.9–59.1]	43.5% [40.9–46.1]
Arkansas	Nov 2016	Medical	43.0 [41.3–44.7]	57.0% [55.3–58.7]
California	Nov 2016	Recreational	45.3 [43.3–47.2]	52.7% [50.8–54.7]
Colorado	Nov 2012	Recreational	35.6 [33.4–37.8]	29.4% [27.2–31.6]
Florida	Nov 2016	Medical	51.4 [49.7–53.1]	48.6% [46.9–50.3]
Maine	Nov 2016	Recreational	39.2 [37.8–40.7]	60.8% [59.3–62.2]
Massachusetts	Nov 2012	Medical	39.7 [38.1–41.3]	30.3% [28.7–31.9]
Michigan	Nov 2018	Recreational	39.6 [38.5–40.8]	60.4% [59.2–61.5]
Missouri	Nov 2018	Medical	40.7 [39.3–42.1]	59.3% [57.9–60.7]
Nevada	Nov 2016	Recreational	28.4 [27.2–29.6]	44.6% [43.4–45.8]
North Dakota	Nov 2016	Medical	35.5 [33.9–37.1]	64.5% [62.9–66.1]
Ohio	Nov 2015	Recreational	60.4 [58.5–62.3]	28.6% [26.7–30.5]
Oklahoma	June 2018	Medical	26.8 [25.5–28.1]	64.2% [62.9–65.5]
Oregon	Nov 2014	Recreational	34.9 [33.4–36.3]	11.1% [9.7–12.6]
South Dakota	Nov 2010	Medical	57.4 [52.4–62.4]	36.6% [31.6–41.6]
Utah	Nov 2018	Medical	48.9 [46.4–51.4]	51.1% [48.6–53.6]
Washington	Nov 2012	Recreational	41.6 [39.6–43.6]	58.4% [56.4–60.4]

^a^For each state election, the mean internet search interest for “cannabis” was obtained from May 1, 2008 through the month before each specific state election

^b^The percent increase in relative internet search interest during the voting month compared to the mean relative internet search interest beginning May 1, 2008 and ending the month prior to voting on the bill

**Fig. 1:**
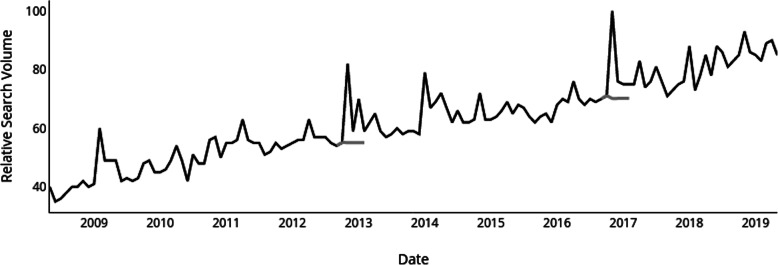
Relative internet search interest in “Cannabis” as a health topic between 2008 and 2019. Legend: Monthly trends in relative internet search volume for “Cannabis” as a health topic in the United States from 2008 to 20,019 was measured using Google Trends. Observed internet search trends (black line) vs. expected forecasts (gray line) calculated using an ARIMA model following the presidential elections of 2012 and 2016. The spike in searches for cannabis during January of 2014 is most likely due to the start of the first legal recreational cannabis sales in Colorado

For the US, observed internet search interest during each Presidential Election was 26.9% [95% CI, 18–35%] greater than expected in 2012 and 29.8% [95% CI, 21–39%] greater than expected in 2016 (Fig. 1). The mean relative internet search volume 6 months before the 2012 Presidential Election was 55.8 [95% CI, 54.4–57.2], the relative internet search volume during election month was 82, and the mean relative internet search volume 6 months after the election was 62.3 [95% CI, 57.7–67.0]. The mean relative internet search volume 6 months before the 2016 Presidential Election was 69.7 [95% CI, 68.6–70.8], the relative internet search volume during election month was 100, and the mean relative internet search volume 6 months after the election was 76.3 [95% CI, 72.8–79.8]. Of the five additional issues that were searched as topics, only two showed an increase in internet search interest during each Presidential Election. Climate Change increased by 3.5% [95% CI, − 13-20%] in 2012 and 20.1% [95% CI, 0–40%] in 2016 while Global Warming increased by 1.1% [95% CI, − 19-21%] in 2012 and 4.6% [95% CI, − 6-15%] in 2016. Table 2 presents the results of all additionally searched issues during the Presidential Elections.

**Table 2:** Public internet search interest in major public issues during the 2012 and 2016 presidential elections

Education	Increase [95%, CI]
November 2012	−4.0% (− 11–3)
November 2016	2.0% (−3–7)
Gun control	
November 2012	5.6% (0–11)
November 2016	−0.8% (− 25–14)
Climate change	
November 2012	3.5% (− 13–20)
November 2016	20.1% (0–40)
Global warming	
November 2012	1.1% (− 19–21)
November 2016	4.6% (− 6–15)
Abortion	
November 2012	−2.0% (− 11–7)
November 2016	2.2% (− 6–11)
Cannabis	
November 2012	26.9 (18–35)
November 2016	29.8 (21–39)

Google Trends provided relative internet search volume data for the election month and the mean internet search volume for the election month was compared against the forecasted autoregressive integrated moving average (ARIMA). The ARIMA model provides a forecast or predicted relative internet search volume in the event the election did not occur

**Fig. 2:**
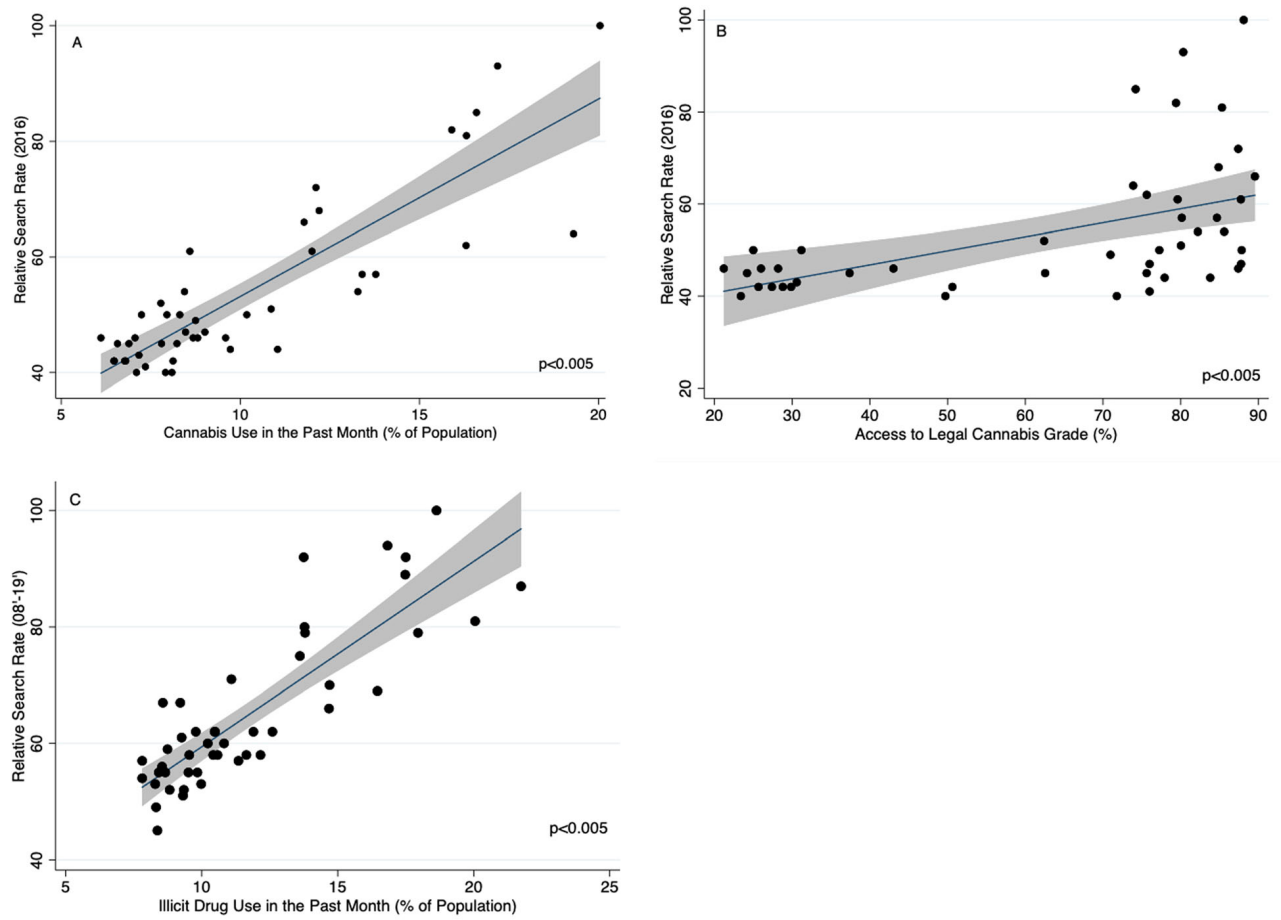
State-Level Associations Between Cannabis Use, Illicit Drug Use, Access To Legal Cannabis and Relative Internet Search Rates for Cannabis. Legend: State-level associations between relative internet search rates for cannabis and **a** cannabis use in the past month (coeff (95% CI), 3.4 (2.8–4.0)), **b** access to legal cannabis grade (coeff (95% CI), 0.31 (0.15–0.46)) and **c** illicit drug use in the past month (coeff (95% CI), 3.1 (2.5–3.7)). Relative internet search rates for cannabis were obtained using Google Trends. All cannabis and illicit drug use statistics were obtained from the National Survey on Drug Use and Health (National Institute on Drug Abuse 2019). Each state’s Access to Legal Cannabis Grade was obtained from American’s for Safe Access (Reports [Internet] 2020). The black dots represent each state, the gray line represents the 95% CI and the blue line is the regression line. All associations were made using a linear regression for the beta coefficients in Stata 15.1

In 2016, significant state-level findings (Fig. 2) included an increase in relative internet search rates for cannabis in states with higher usage rates of cannabis in the past month (Coeff (95% CI), 3.4 (2.8–4.0)). Relative internet search rates for cannabis from 2008 to 2019 were also associated with increased cannabis usage in the past month (Coeff (95% CI), 3.1 (2.5–3.7)). States with higher access to legal cannabis grades were associated with higher relative search volumes for cannabis (Coeff (95% CI), 0.31 (0.15–0.46)). Similar to cannabis, relative internet searches for “climate control” and “global warming” were associated with significant associations between “cannabis use in the past month”, “drug use in the past month excluding cannabis”, and access to legal cannabis grade (Table 3).

**Table 3:** Association between topic-specific internet search rates and state-level drug use characteristics

Internet search term	State-level drug use characteristic	Coefficient (***p*** value)	95% CI	R^2
**Education**	cannabis use in the past month (18+ years old)	−0.12 (0.77)	− 1.0 - 0.74	0.00
	drug use in the past month (excluding cannabis use) (18+ years old)	1.1 (0.73)	−5.6 - 7.8	0.00
	access to legal cannabis	−0.06 (0.37)	−0.19 - 0.07	0.02
**Gun control**	cannabis use in the past month (18+ years old)	1.2 (0.01)	0.31–2.1	0.14
	drug use in the past month (excluding cannabis use) (18+ years old)	4.0 (0.28)	−3.4 - 11.3	0.03
	access to legal cannabis	0.01 (0.91)	−0.14 - 0.15	0.00
**Climate change**	cannabis use in the past month (18+ years old)	3.2 (< 0.005)	2.5–4.0	0.60
	drug use in the past month (excluding cannabis use) (18+ years old)	14.8 (< 0.005)	6.1–23.5	0.20
	access to legal cannabis	0.27 (< 0.005)	0.09–0.44	0.18
**Global warming**	cannabis use in the past month (18+ years old)	2.1 (< 0.005)	1.3–2.9	0.41
	drug use in the past month (excluding cannabis use) (18+ years old)	10.0 (0.006)	3.0–17.0	0.15
	access to legal cannabis	0.17 (0.02)	0.03–0.31	0.12
**Abortion**	cannabis use in the past month (18+ years old)	−0.88 (0.006)	−1.5 - -0.26	0.15
	drug use in the past month (excluding cannabis use) (18+ years old)	−2.44 (0.34)	−7.6 - 2.7	0.02
	access to legal cannabis	−0.09 (0.7)	−0.19 - 0.01	0.07
**Cannabis**	cannabis use in the past month (18+ years old)	3.4 (< 0.005)	2.8–4.0	0.74
	drug use in the past month (excluding cannabis use) (18+ years old)	17.4 (< 0.005)	9.8–25.0	0.33
	access to legal cannabis	0.31 (< 0.005)	0.15–0.46	0.26

Internet search rates were obtained using Google Trends for 2016 and these rates were observed for associations to the National Survey on Drug Use and Health for 2016–17 statistics (National Institute on Drug Abuse 2019) and access to legal cannabis grades using a linear regression for the beta coefficient values in Stata 15.1. State-Level Drug Use Characteristics were from the National Survey on Drug Use and Health (National Institute on Drug Abuse 2019). Access to legal cannabis was based upon the American’s for Safe Access state cannabis grade (Reports [Internet] 2020)

## Discussion

This study showed that US relative internet search interest in cannabis as a health topic increased dramatically during election months when cannabis legislation was on state ballots. Of the 18 total states analyzed, the mean percent increase for relative search volume during the voting month was 48.3%. In addition, this search volume also increased during the 2012 and 2016 Presidential Election months by 26.9 and 29.8%, respectively. Of the five selected control policies, only two showed an increase in internet search volume during both Presidential Elections and neither exceeded the internet search increase of cannabis. These results may indicate the growing awareness of cannabis in the US and mark a possible target for the timely dissemination of evidence-based information regarding cannabis and its usage/side-effects during future elections. In addition, this study’s analysis highlighted significant state-level associations with cannabis internet search rate increases. For example, states with higher increases in relative internet search rates for cannabis were associated with a higher incidence of cannabis and past month illicit drug use except cannabis during the year of 2016. Furthermore, increases in relative internet search rates for cannabis from 2008 to 2019 were also associated with increased cannabis usage in the past month and higher access to legal cannabis grades. Thus, policymakers and state legislators may use this information to target high-risk states during election periods and possibly prevent adverse events that are related to excessive cannabis and illicit drug use through marketing and educational tactics through the dissemination of evidence-based resources. Additionally, we underscore that results from these analyses are correlational rather than causal and should be interpreted as such.

A study conducted by Linkov et al. (2010) in 2010, investigated public interest in disasters using Google Trends to determine when people are most interested in such global events. This study retrospectively examined four events that may have had a major effect on public interest by searching for the terms “tsunami”, “hurricane”, “H1N1”, and “earthquake” during a 6 year time period from 2004 to 2010 and then examined the associated Google Trends interest curves. The results of this study showed that despite differing levels of interest, all four events showed an identical interest curve increasing after the event and decreasing shortly after the spike. The authors concluded that since the interest level curve increases shortly after the events, information must be disseminated quickly in the associated time-frame to allow for proper education regarding the searched topic. Our results show a similar curve to the disasters searched where there is an abrupt increase in the interest level and a sharp decay shortly after. Furthermore, this information provides additional evidence of an opportune time for the dissemination of evidence-based information regarding cannabis during elections.

Based on these results, we can expect public interest in cannabis to spike again prior to the Presidential Election in 2020 and future elections. With this expected spike in interest, there is an imperative need for accurate and evidenced-based information regarding cannabis to be available to the public during these periods of increased public interest. Consequently, the implications of this study are important to physicians since they will likely receive an increased volume of questions relating to cannabis and its therapeutic uses during election season from interested patients. However, one study found that half of the primary care physicians surveyed were not ready or did not want to answer questions regarding cannabis (Philpot et al. 2019). Given the likely increase in cannabis internet search interest in 2020 and the controversy that surrounds cannabis, physician awareness and education is paramount for providing evidence-based recommendations to these patients.

To facilitate awareness, we detail a few recommendations the medical community may consider to enable physicians to provide the highest quality care. First, we recommend a cannabis repository of evidence-based information be established for physicians and widely promoted. This repository should be easily searchable and free of stakeholder bias to inform provider and patient medical decisions. Second, we recommend educating physicians on the inherent conflicts of interest within cannabis legislation and the difficulties in conducting clinical trials to provide evidence for its effectiveness (Hill 2019; Bowling and Glantz 2019). Lastly, given that many resident and fellow physicians report having little knowledge about medicinal cannabis, we suggest that efforts be made to educate and train future physicians on the risks and benefits of cannabis (Evanoff et al. 2017). One avenue to this education could be implementing cannabis education into the curriculum of medical schools and post-graduate residency programs. We feel these actions will better prepare physicians to provide evidence-based guidance to their patients on issues relating to medicinal cannabis despite little evidence amongst the literature.

Regarding the strengths of our study, we used previously published methodology for measuring public interest in a specific topic as well as forecasting ARIMA models (Ayers et al. 2017; Ayers et al. 2019; Torgerson et al. 2019). We also used data from a validated, large-scale, nationally representative survey (NSDUH) to make associations with cannabis search volume. However, we are aware that while Google is the most commonly used internet search engine it may not be a complete representation of public internet activity in its entirety. We also must note that the state-level associations are correlations and not causative and hence may be subject to unforeseen confounding.

## Conclusion

In summary, our investigation showed that US relative internet search interest in cannabis increased dramatically when cannabis legislation was on state ballots during election months. Based on these results, we expect public interest in cannabis will spike prior to the Presidential election in 2020 which could provide an opportune time for the dissemination of evidence-based information through online platforms. Consequently, the physician implications are important considering the likely increase in volume of questions relating to cannabis and its therapeutic uses during election season. We recommend establishing a cannabis repository of evidence-based information, providing physician education, and a dosing guide be created to enable physicians to provide high quality care around the issue of cannabis.

## Data Availability

Data is available upon request.

## Abbreviation

ARIMA: Autoregressive integrated moving average
